# Administration of Epidermal Growth Factor (EGF) and Basic Fibroblast Growth Factor (bFGF) to Induce Neural Differentiation of Dental Pulp Stem Cells (DPSC) Isolates

**DOI:** 10.3390/biomedicines11020255

**Published:** 2023-01-18

**Authors:** Keegan Lott, Paris Collier, Marc Ringor, Katherine M. Howard, Karl Kingsley

**Affiliations:** 1 School of Medicine, University of Nevada-Las Vegas, 1700 W. Charleston Boulevard, Las Vegas, NV 89106, USA; 2 Department of Biomedical Sciences, School of Dental Medicine, University of Nevada-Las Vegas, 1001 Shadow Lane, Las Vegas, NV 89106, USA

**Keywords:** dental pulp stem cell (DPSC), mesenchymal stem cell (MSC), tissue regeneration

## Abstract

The aging populations in many countries have developed many chronic illnesses and diseases, including chronic neurologic conditions such as Parkinson’s and Azheimer’s diseases. Many new lines of research and treatment are focusing on the potential for neurologic regeneration using mesenchymal stem cells (MSCs) in the rapidly growing field of regenerative medicine. This may include dental pulp stem cells (DPSCs), which have recently been demonstrated to produce neuronal precursors. Based upon this evidence, the primary aim of this study was to determine if the growth factors used in MSC-based studies are sufficient to induce neuronal differentiation among DPSCs. Using an existing biorepository, *n* = 16 DPSC isolates were thawed and cultured for this study, which revealed several subpopulations of rapid-, intermediate-, and slowly dividing DPSCs. Administration of epidermal growth factor (EGF) and basic fibroblast growth factor (bFGF) were sufficient to induce differential changes in growth and viability mainly among some of the rapidly growing DPSCs (*n* = 4). These phenotypic changes included expression of neural differentiation markers including Sox1, Pax6 and NF-M, which were observed only among those DPSC isolates not expressing early odontoblast-specific biomarkers such as ALP and DSPP. Future studies will be needed to confirm if these methods are sufficient to induce consistent and reliable induction of DPSCs towards neuronal specific differentiation.

## 1. Introduction

With rapidly aging populations, many developed countries have now recognized that neurologic disorders are a major cause of disease and disability in the world [1,2]. Neurologic disorders include a vast array of conditions, such as Alzheimer’s and Parkinson’s diseases, multiple sclerosis, epilepsy, dementias, as well as headache- or migraine-related neuropathies [3]. Recent estimates have suggested that these conditions are among the largest contributors to disability-adjusted life years (DALYs) in the US and around the world [4,5].

Although some progress has been made in recent years towards developing therapies and treatments for these wide-ranging disorders, the growing burden of neurologic diseases has produced new lines of research and cell-based therapies involving mesenchymal stem cells (MSCs) [6,7]. Many of these studies have focused on the potential for MSCs in treatments for traumatic brain and spinal cord injury [8,9]. However, MSC-based therapies are now being developed for a wide range of disorders, including epilepsy, Parkinson’s disease, motor neuron disease and multiple sclerosis [10,11,12,13].

Previous research studies have shown that mesenchymal stem cells, such as dental pulp stem cells or DPSCs, can differentiate into neuron-like cells when cultured with vascular endothelial growth factor (VEGF), epithelial growth factor (EGF), and basic fibroblast growth factor (bFGF) [14,15]. In fact, some evidence has suggested that other types of MSCs, such as bone marrow stem cells, may be less effective at neuronal differentiation compared to DPSCs [16,17]. As evidence continues to grow demonstrating DPSCs’ potential in neurologic and neuronal research, more detailed studies are needed to provide standardization for the most effective methods and protocols that guide DPSC differentiation and growth [18,19].

Some guidance regarding DPSC protocols has been developed for isolation, characterization, and expansion of DPSCs in vitro [20,21,22]. Research from this group has uncovered some of the biomarkers and microRNA expression associated with increased viability and proliferation following cryopreservation, although functional studies about neurogenic differentiation potential have not yet been conducted [23]. However, much less evidence exists to facilitate this research specifically for neural differentiation [24,25]. 

For example, new evidence has suggested that bFGF may act in conjunction with EGF in guiding DPSCs towards neuronal differentiation [26]. In addition, these studies have demonstrated that bFGF and EGF may induce temporal- and biomarker-specific events that regulate the process of DPSC neuronal differentiation through extracellular-signal-regulated kinase 1 and 2 (ERK 1/2) and protein kinase B (Akt) pathways [26,27]. However, these studies also suggest that much remains unknown about the propagation and induction of DPSCs towards neuronal differentiation [28]. Based upon this information, the specific aims of this study are to determine if basic fibroblast growth factor (bFGF) and/or epidermal growth factor (EGF) are sufficient to induce neural differentiation of dental pulp stem cells (DPSCs). 

## 2. Materials and Methods

### 2.1. Study Approval

This study utilized DPSCs from an existing repository at the University of Nevada, Las Vegas (UNLV). The Institutional Review Board (IRB) and Office for the Protection of Research Subjects (OPRS) reviewed and approved this study under “Retrospective analysis of dental pulp stem cells (DPSC) from the University of Nevada, Las Vegas (UNLV) School of Dental Medicine (SDM) pediatric and clinical population” as Protocol 171612-1 on 21 February 2021. 

The original study to collect human DPSCs and create the repository was approved under protocol OPRS#0907-3148 “Isolation of Non-Embryonic Stem Cells from Dental Pulp” on 5 February 2010. In brief, each of the study’s participants voluntarily participated and were asked to provide informed consent. The inclusion criteria required that all participants be patients of record at the UNLV School of Dental Medicine (SDM) clinic that were already scheduled for extractions of vital, permanent third molar teeth as a routine part of an on-going orthodontic therapy treatment plan. Exclusion criteria included any patient or guardian that declined to participate, any person not a patient of record at UNLV-SDM, and any patient requiring tooth extraction for alternative reasons, including periodontal disease, fractures, or extreme dental decay.

All DPSC isolates were originally passed in cell culture for a minimum of ten (*n* = 10) passages before the cryopreservation protocol, which included 10% dimethyl sulfoxide (DMSO)-containing media with fetal bovine serum (FBS). For the current study, each DPSC isolate was thawed on ice and then centrifuged for five minutes at 2100 × relative centrifugal force (RCF) to remove the DMSO-containing media prior to resuspension in cell culture media. Prior studies from this group established the appropriate media for DPSC culturing, which included Dulbecco’s modified eagle’s medium or DMEM, DMEM:Nutrient Mixture F12, Roswell Park Memorial Institute or RPMI, alpha-Minimum Essential Media or MEM (supplemented with 10% FBS and 1% penicillin–streptomycin) all from Gibco (Waltham, MA, USA) [14,23].

### 2.2. Cell Culture

After thawing and resuspension, all DPSC isolates (*n* = 16) were cultured using the appropriate media within tissue-cultured-treated 25 cm^3^ flasks and placed inside a biosafety level (BSL)-2 incubator supplemented with 5% CO2 at 37 °C. Each individual DPSC isolate was previously categorized as exhibiting a rapid doubling time (rDT) of 2–3 days, an intermediate doubling time (iDT) of 5–6 days, or a slow doubling time (sDT) of 10–14 days [14,23]. These characteristics were confirmed upon thawing, which included the following (Table 1).

### 2.3. Viability

Measurement of cell viability was performed using the trypan blue exclusion assay. In brief, 20 µL of each DPSC isolate media was removed and mixed with 0.4% Trypan Blue Solution from Fisher Scientific (Fair Lawn, NJ, USA). The solution was mixed thoroughly and analyzed for cell viability using a TC 20 automated cell counter from BioRad (Hercules, CA, USA). Automated cell count values for total number of cells, total number of live cells, and percentage of live cells were obtained and recorded. Cell viability was assessed every three days and throughout all experimental assays.

### 2.4. Proliferation and Growth Assays

For each experiment, cells were plated in 96-well assay plates at equal concentrations of 1.2 × 10^5^ cells/mL and were allowed to proliferate for one (24 h), two (48 h) or three days (72 h). At the end of each experimental timepoint, cells were fixed through the addition of 50 µL 10% buffered formalin from Fisher Scientific (Fair Lawn, NJ, USA). After 24 h, the formalin was aspirated and the cells stained with 100 µL 1% gentian violet solution from Fisher Scientific (Fair Lawn, NJ, USA). The stain was aspirated and cells were then washed with 100 µL of 1X phosphate-buffered saline (PBS) from Fisher Scientific (Fair Lawn, NJ, USA). After aspiration of the 1X PBS, each assay plate was processed using the ELx808 microplate reader from Eppendorf (Hamburg, Germany) and analyzed using a wavelength of 630 nm as previously described [29,30]. 

### 2.5. Growth Factor Treatment

Growth factors for the experimental treatment of DPSC isolates included Epidermal growth factor or EGF (catalog #PHG0311) and basic fibroblast growth factor or bFGF (catalog #13256029) were obtained from Gibco (Waltham, MA, USA). Concentrations of EGF at 20 ng/mL and bFGF at 20 ng/mL were used in the experimental assays, which were consistent with concentrations utilized in other studies of DPSC neuronal differentiation [31,32]. Negative controls for each experimental assay involved the use of 1X PBS in place of the addition of growth factors into the cell culture media. 

### 2.6. RNA Extraction

To evaluate biomarker expression among the DPSC isolates, RNA was extracted using the phenol:chloroform method, as previously described [23,29,30]. In brief, 500 µL of TRIzol reagent from Fisher Scientific (Fair Lawn, NJ, USA) was applied to an equal volume of cell suspension (500 µL) and mixed prior to the addition of 200 µL of molecular grade chloroform from Sigma Aldrich (St. Louis, MO, USA). Following incubation on ice, samples were centrifuged at 12,000 × relative centrifugal force (RCF) at 4 °C in a 5424 Microcentrifuge from Eppendorf (Hamburg, Germany). The upper aqueous RNA-containing phase was removed and combined with an equal volume of molecular grade isopropanol from Sigma Aldrich (St. Louis, MO, USA) and incubated on ice for ten minutes to precipitate the nucleic acids. Each sample was centrifuged again for 10 min and the supernatant was removed prior to washing with 70% ethanol from Sigma Aldrich (St. Louis, MO, USA). Following a final centrifugation for five minutes, the supernatant was removed and the RNA-containing pellet was resuspended in 100 µL of nuclease-free water from Fisher Scientific (Fair Lawn, NJ, USA). Analysis of RNA isolation was completed using a NanoDrop 2000 spectrophotometer from Fisher Scientific (Fair Lawn, NJ, USA) to evaluate the concentration and purity of the RNA. Each sample was subsequently stored at −20 °C.

### 2.7. qPCR Biomarker Screening

All samples of extracted DPSC RNA were converted into cDNA using an ABgene Reverse-iT One-Step RT-PCR kit from Fisher Scientific (Fair Lawn, NJ, USA) and the Mastercycler gradient thermal cycler from Eppendorf (Hamburg, Germany). In brief, each reaction contained 2X Reddy Mix (12.5 µL of RT-PCR Master mix), DPSC RNA (1.0 ug), forward and reverse random primers (1.5 µL) from Invitrogen (Waltham, MA, USA), RTase blend (1.0 µL), and nuclease-free water standardize volume of each reaction to 25 µL. Thermocycler settings were obtained from the manufacturer recommendations, which included 47 °C for 30 min for reverse transcription and a final extension at 72 °C for five minutes. 

qPCR screening was performed on each DPSC sample with the SYBR green real-time PCR Master Mix from ThermoFisher Scientific (Fair Lawn, NJ, USA) using the manufacturer recommended protocol. Briefly, each reaction contained 2X SYBR Green PCR Master Mix (12.5 µL), forward and reverse primers specific for each designated target (1.5 µL), cDNA (1.0 µL of final dilution to 10 ng/µL) and nuclease-free water (8.5 µL). Real-time PCR was performed using QuantStudio 5 Real-Time PCR system from Fisher Scientific (Fair Lawn, NJ, USA) and settings that included 10 min at 95 °C for initial activation, 15 s at 95 °C for denaturation and 40 cycles of annealing and extension at the appropriate primer pair-specific temperature for one minute. Stem cell positive control markers CD90 and CD105, as well as the absence of CD45, were confirmed in accordance with the guidelines specified by the International Society for Cellular Therapy (ISCT), as previously described [14,23,33]. Primers were synthesized using SeqWright from ThermoFisher Scientific (Fair Lawn, NJ, USA) (Table 2).

### 2.8. Statistical Analysis

All parametric data analyses involving growth and viability assay were exported to Microsoft Excel (XLS) for analysis. Differences between experimental conditions were measured using two-tailed Student’s *t*-tests, which are the appropriate test for parametric analysis of continuous data. Any statistically significant differences were then subsequently verified using analysis of variance (ANOVA), due to the possibility of error involved with analysis of multiple two-way *t*-tests. Significance levels were set at alpha (ꭤ) = 0.05.

## 3. Results

**Figure 1:**
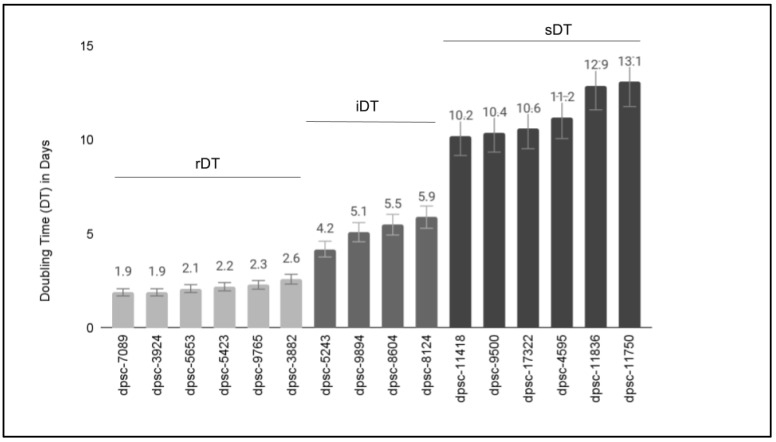
Growth rates of DPSC isolates in culture. Doubling times (DT) for *n* = 6 DPSC isolates were confirmed as rapid or rDT (average 2.1 days), *n* = 4 as intermediate or iDT (average 5.2 days), and *n* = 6 as slow or sDT (average 11.4 days).

The available DPSC isolates were placed into a culture to establish and confirm previous reports of growth rates (Figure 1). More specifically, these results confirmed that six DPSC isolates (dpsc-3882, dpsc-3924, dpsc-5423, dpsc-5653, dpsc-7089, dpsc-9765) exhibited rapid doubling times or rDT between 1.9 and 2.6 days (average 2.1 days). Four DPSC isolates (dpsc-5243, dpsc-8604, dpsc-9894, dpsc-8124) exhibited intermediate doubling times or iDT between 4.2 and 5.9 days (average 5.2 days). Finally, the remaining six DPSC isolates (dpsc-4595, dpsc-9500, dpsc-11418, dpsc-11750, dpsc-11836, dpsc-17322) exhibited slow doubling times or sDT between 10.2 and 13.1 days (average 11.4 days).

**Figure 2:**
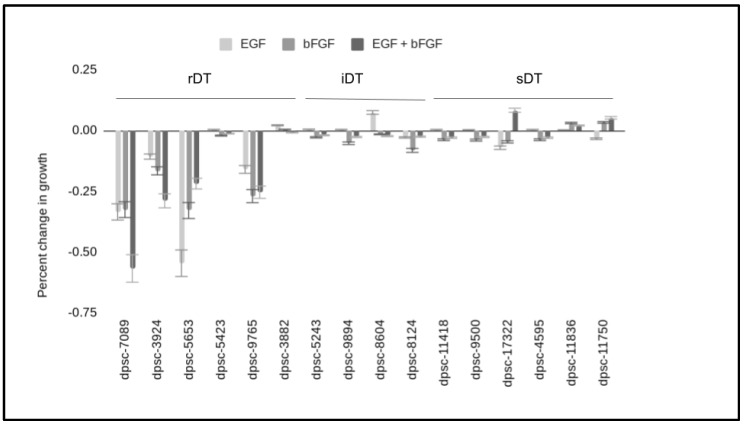
Experimental DPSC proliferation assays. DPSC plated into three-day 96-well assays revealed EGF and bFGF (alone and in combination) significantly altered growth in four rDT DPSC isolates (dpsc-7089, dpsc-3924, dpsc-5653, dpsc-9765) compared with baseline controls, *p* = 0.0001. Only minor, non-significant changes in growth were observed with iDT and sDT DPSC isolates, *p* > 0.05.

To determine any effects of these growth factors on the DPSC isolates, 96-well assays were performed with and without EGF and bFGF—both alone and in combination (Figure 2). These data demonstrated that most of the rDT (*n* = 4/6) DPSC isolates exhibited strong changes in growth following the administration of EGF, bFGF or both. More specifically, the addition of EGF, bFGF and the combination of EGF with bFGF significantly altered the growth of dpsc-7089 (−33.5%, −32.5%, −52.7%), dpsc-3924 (−10.7%, −16.6%, −29.9%), dpsc-5653 (−54.5%, −32.9%, −21.8%), and dpsc-9765 (−15.9%, −26.9%, = 25.3%) compared with baseline controls, *p* = 0.0001. Although minor changes in growth were observed with iDT and sDT DPSC isolates, none of these were statistically significant, *p* > 0.05. 

**Figure 3:**
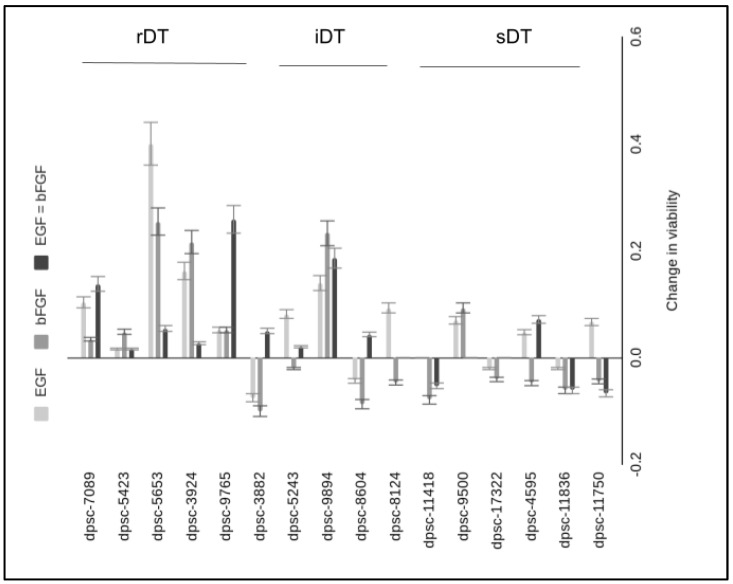
Change in DPSC viability induced by EGF and bFGF administration. Significant increases in viability were observed among four DPSC isolates—including dpsc-7089 (10.3%, 3.4%, 13.8%), dpsc-3924 (16.2%, 21.6%, 2.7%), dpsc-5653 (40.0%, 25.5%, 5.5%), dpsc-9765 (5.1%, 5.1%, 25.8%). Additionally, changes in viability were found with the iDT isolate dpsc-9894 and EGF, bFGF, and combination specific increases of 13.9%, 23.2%, and 18.6%, respectively.

In addition, any effects on viability induced through EGF and bFGF administration were also evaluated (Figure 3). These results demonstrated that the majority of significant increases in DPSC viability were observed among the rDT isolates. More specifically, the four DPSC isolates with the most significant changes in growth also exhibited the most significant changes in viability in response to EGF, bFGF or the combination of both compared with baseline—including dpsc-7089 (10.3%, *p* = 0.038; 3.4%, *p* = 0.12 13.8%, *p* = 0.029), dpsc-3924 (16.2%, *p* = 0.026; 21.6%, *p* = 0.021; 2.7% *p* = 0.14), dpsc-5653 (40.0%, *p* = 0.001; 25.5%, *p* = 0.016; 5.5%, *p* = 0.08), dpsc-9765 (5.1%, *p* = 0079; 5.1%, *p* = 0.079; 25.8%, *p* = 0.019). Although no significant change in growth was found, another notable change in viability was observed among the iDT isolates dpsc-9894 with EGF, bFGF, and combination specific increases of 13.9%, *p* = 0.029; 23.2%, *p* = 0.018; and 18.6%, *p* = 0.031, respectively.

**Figure 4:**
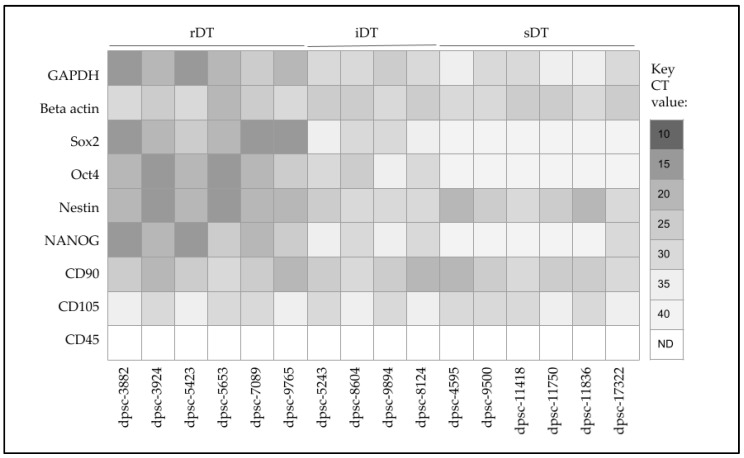
qPCR screening of MSC and DPSC biomarkers. All isolates expressed positive controls (GAPDH, beta actin), as well as ISCT positive controls for DPSC (CD90, CD105). sDT and iDT isolates expressed MSC biomarkers (Sox2, Oct4, Nestin, NANOG). sDT isolates expressed Nestin but not Sox2, Oct4 or NANOG (except dpsc-17322).

Screening of extracted RNA was performed to determine the baseline biomarker expression for each DPSC isolate (Figure 4). These data demonstrated that the positive control metabolic marker glyceraldehyde 3-phosphate dehydrogenase (GAPDH) and structural marker beta actin were expressed (but variable) in all DPSC isolates. In addition, the ISCT positive control markers CD90 and CD105 were also expressed among all DPSC isolates, although expression levels were also variable. None of the DPSC isolates expressed the negative control ISCT marker CD45.

Expression of the MSC biomarkers Sox2, Oct, Nestin and NANOG was observed among all the rDT (dpsc-3882, dpsc-3924, dpsc-5423, dpsc-5653, dpsc-7089, dpsc-9765) and iDT (dpsc-5243, dpsc-8604, dpsc-9894, dpsc-8124) DPSC isolates. Although Nestin was observed among all sDT DPSC isolates (dpsc-4595, dpsc-9500, dpsc-11418, dpsc-11750, dpsc-11836, dpsc-17322), NANOG was only observed in dpsc-17322. In addition, Sox2 and Oct4 were not observed in any of the sDT DPSC isolates.

**Figure 5:**
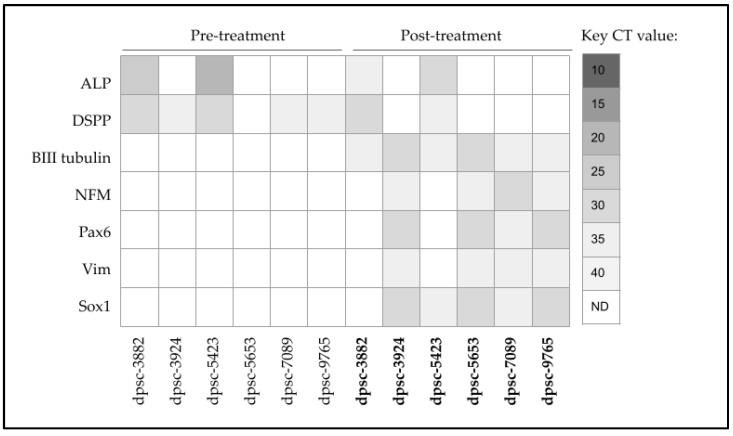
Screening of DPSC mRNA expression pre- and post-treatment. DPSC markers ALP and DSPP were expressed in both EGF-bFGF non-responsive lines dpsc-3882 and dpsc-5423. Expression of neural differentiation markers ꞵIII tubulin, NFM, Pax6, Vim, and Sox1 were observed among the EGF-bFGF responsive lines dpsc-3924, dpsc-5653, dpsc-7089 and dpsc-9765.

To more closely evaluate the differential changes induced by EGF and bFGF treatment on the rDT DPSC isolates, DPSC differentiation markers alkaline phosphatase (ALP) and dentin sialophosphoprotein (DSPP) were evaluated (Figure 5). These data demonstrated that the two non-responsive rDT DPSC isolates (dpsc-3882, dpsc-5423) both expressed ALP and DSPP. Only modest expression of DSPP was observed among dpsc-3924, dpsc-7089 and dpsc-9765 with no expression of ALP dpsc-3924, dpsc-5653, dpsc-7089 and dpsc-9765. 

Following combination treatment with EGF and bFGF, evaluation of the neural differentiation markers demonstrated all of the rDT DPSC isolates produced the early differentiation marker beta III tubulin, although this was more pronounced among the EGF- and bFGF-growth responsive isolates, dpsc-3924 and dpsc-5653. Expression of neurofilament M protein (NFM) was present and more modest in all rDT isolates except dpsc-7089. In addition, expression of the early neural development signaling mediators Pax6 and Sox1 were observed in all rDT isolates, with more robust expression observed among the most responsive DPSC isolates, dpsc-3924, dpsc-5653, and dpsc-9765. Finally, expression of Vimentin (Vim) was detectable in all rDT DPSC isolates in the post-treatment group. 

**Figure 6:**
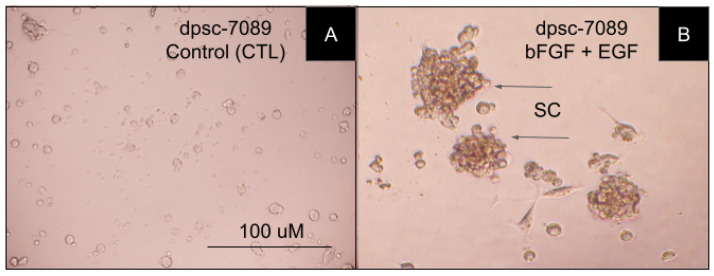
Microscopy of DPSC rDT isolate dpsc-7089 under control and experimental conditions. (**A**). Adherent image of non-treated control assay of dpsc-7089. (**B**). Significant induction of spherical clumping (SC) demonstrates morphologic changes induced by bFGF and EGF combination treatment in dpsc-7089 cells.

Cellular microscopy was used to further examine any phenotypic changes associated with bFGF and EGF treatment of DPSC isolates (Figure 6). More specifically, non-treated DPSC in the non-treated or control wells, such as the bFGF- and EGF-responsive rDT DPSC isolate dpsc-7089 (Figure 6A). In comparison, the treated cells were also evaluated, including dpsc-7089 under bFGF and EGF combination treatment, which demonstrated significant differences in cellular morphology, including spherical clumping (SC)—observed in other populations of MSCs during neuronal differentiation and induction (Figure 6B). 

## 4. Discussion

The primary goal of this project was to determine if the administration of basic fibroblast growth factor (bFGF) and/or epidermal growth factor (EGF) are sufficient to induce neural differentiation of dental pulp stem cells (DPSCs). These results demonstrated that both EGF and bFGF are sufficient to induce phenotypic changes and biomarker expression, but these changes were not uniform across all DPSC isolates. Moreover, the analysis of these data have revealed that a combination of several distinct factors and biomarkers may be needed to determine DPSC responsiveness, similar to the results of other studies regarding DPSC differentiation for bone and cartilage repair [34,35,36,37].

One of the most important findings of this study is that differentiation potential among the DPSC isolates may be closely associated with proliferation rate, confirming observations reported in other studies that the most rapidly dividing DPSC isolates may be the most responsive to growth factor administration and differentiation induction [38,39]. In fact, previous studies from this group have made similar observations about DPSC responsiveness and growth rate, which appear to be consistent over time and among varied isolates from distinct individuals [25,26,27,28]. However, this study also revealed that within this group of rapidly dividing DPSC isolates, heterogeneity among additional factors and biomarkers appears to significantly influence growth factor responsiveness and differentiation potential, as previous studies have also demonstrated [14,40]. 

For example, although all of the rapidly dividing isolates expressed the pluripotency markers Sox-2, Oct-4, Nestin and NANOG as reported in previous DPSC studies, not all of these were responsive to bFGF or EGF administration [41,42,43]. The results of this current study found that additional factors, including the presence of ALP and DSPP (even at relatively low expression levels) may hinder the ability of DPSC isolates to respond to these growth factors, a phenomenon that has been observed in other studies of DPSC differentiation [44,45,46]. In fact, this type of negative regulation with specificity towards only limited lineage potential may be more common than previously recognized and the factors that regulate these mechanisms are important to further research progress in this field [47,48].

This study also limited the focus of DPSC neural responsiveness to EGF and bFGF, which has been the subject of previous DPSC neural differentiation studies [15,31,32]. Additional methods for neural regulation and induction in both MSCs and DPSCs have also demonstrated some limited potential, such as the use of specific extracellular matrix (ECM) molecules to create environmental cues and stimulate microenvironment-specific signaling pathways [49,50]. Future research in this area might benefit from investigating a combination of specific ECM with these growth factors to determine if this type of synergistic approach can yield more effective differentiation than either protocol alone [51]. 

Although this study revealed some important regulatory mechanisms between DPSC responsiveness to growth factor stimulation, there are several limitations that should be considered when evaluating these results. First, this is a retrospective study of previously collected DPSCs stored in a biorepository, which may be a confounding variable in this type of research due to the uncontrolled and variable effects of long-term cryopreservation on DPSC viability and differentiation potential [52,53]. In addition, due to financial constraints and other time limitations, only DPSCs from this institution were available for analysis, which suggests that other studies investigating these factors and DPSC responsiveness will be needed to confirm if these results are consistent across multiple and varied DPSC isolates as has been demonstrated in other studies of MSCs [54]. Finally, other novel approaches, such as harvesting MSC secreted factors, including microRNAs and other extracellular vesicles, were not possible with the resources available for this study but may provide other potential avenues for further investigation into DPSC neural differentiation [55,56].

Studies are now beginning to demonstrate the clinical potential for MSCs to improve outcomes in neurological conditions, such as Huntington’s disease [57]. In fact, preclinical studies of DPSC therapies have now demonstrated significant improvements in neurologic function to promote recovery following ischemic stroke [58]. These exciting developments may represent the leading edge of DPSC-specific treatments that may provide significant improvements for a broad range of neurologic conditions that require regenerative therapies in the near future [59].

## 5. Conclusions

This study demonstrated that EGF and bFGF are sufficient to induce phenotypic plasticity and differentiation-specific changes among DPSCs. However, this study also identified several factors that may also regulate the responsiveness of these DPSC isolates, although more research will be needed to determine if growth rate and biomarker expression are consistent factors that determine responsiveness among other populations of extracted DPSCs. These results add to the evidence that DPSCs may be an important component of developing treatments and therapies for the growing number of neurologic and neurodegenerative diseases facing our aging populations.

## Figures and Tables

**Table 1:** DPSC isolate characteristics.

DPSC Category	DT (Days)	DPSC Isolate
rapid doubling time (rDT) DPSC isolates	2–3 days	dpsc-3882, dpsc-3924, dpsc-5423, dpsc-5653, dpsc-7089, dpsc-9765
intermediate doubling time (iDT) DPSC isolates	5–6 days	dpsc-5243, dpsc-8604, dpsc-9894, dpsc-8124
slow doubling time (sDT) DPSC isolates	10–12 days	dpsc-4595, dpsc-9500, dpsc-11418, dpsc-11750, dpsc-11836, dpsc-17322

**Table 2:** Validated primer sequence table.

Primer Name	Sequence
Glyceraldehyde 3-phosphate dehydrogenase (GAPDH) GAPDH forward primer	5′-ATC TTC CAG GAG CGA GAT CC-3′
GAPDH reverse primer	5′-ACC ACT GAC ACG TTG GCA GT-3′
Beta actin forward primer	5′-GTG GGG TCC TGT GGT GTG-3′
Beta actin reverse primer	5′-GAA GGG GAC AGG CAG TGA-3′
Sox-2 forward primer	5′-ATG GGC TCT GTG GTC AAG TC-3′
Sox-2 reverse primer	5′-CCC TCC CAA TTC CCT TGT AT-3′
Nestin forward primer	5′-CGT TGG AAC AGA GGT TGG AG-3′
Nestin reverse primer	5′-TCC TGA AAG CTG AGG GAA G-3′
NANOG forward primer	5′-GCT GAG ATG CCT CAC ACG GAG-3′
NANOG reverse primer	5′-TCT GTT TCT TGA CTG GGA CCT TGT C-3′
Oct-4 forward primer	5′-TGG AGA AGG AGA AGC TGG AGC AAA A-3′
Oct-4 reverse primer	5′-GGC AGA TGG TCG TTT GGC TGA ATA-3′
CD90 forward primer	5′-ATG AAC CTG GCC ATC AGC A-3′
CD90 reverse primer	5′-GTG TGC TCA GGC ACC CC-3′
CD105 forward primer	5′-CCA CTA GCC AGG TCT CGA AG-3′
CD105 reverse primer	5′-GAT GCA GGA AGA CAC TGC TG-3′
CD45 forward primer	5′-CAT ATT TAT TTT GTC CTT CTC CCA-3′
CD45 reverse primer	5′-GAA AGT TTC CAC GAA CGG-3′
Alkaline Phosphatase (ALP) ALP forward primer	5′-CAC TGC GGA CCA TTC CCA CGT CTT-3′
ALP reverse primer	5′-GCG CCT GGT AGT TGT TGT GAG CAT-3′
Dentin sialophosphoprotein (DSPP) DSPP forward primer	5′-CAA CCA TAG AGA AAG CAA ACG CG-3′
DSPP reverse primer	5′-TTT CTG TTG CCA CTG CTG GGA C-3′
Vimentin (Vim) forward primer	5′-GAC GCC ATC AAC ACC GAG TT-3′
Vim reverse primer	5′-CTT TGT CGT TGG TTA GCT GGT-3′
Paired box protein 6 (Pax6) Pax-6 forward primer	5′-TGG GCA GGT ATT ACG AGA CTG-3′
Pax-6 reverse primer	5′-ACT CCC GCT TAT ACT GGG CTA-3′
Beta III tubulin forward primer	5′-GGC CAA GGG TCA CTA CAC G-3′
Beta III tubulin reverse primer	5′-GCA GTC GCA GTT TTC ACA CTC-3′
Neurofilament M protein (NFM) forward primer	5′-GCT CGT CAT TTG CGC GAA TAC-3′
NFM reverse primer	5′-TTT CTG TAC GCA GCG ATT TCT AT-3′
SRY-box transcription factor 1 (Sox-1) forward primer	5′-CAG TAC AGC CCC ATC TCC AAC-3′
Sox-1 reverse primer	5′-GCG GGC AAG TAC ATG CTG A-3′

## Data Availability

The data presented in this study are available on request from the corresponding author.
